# Impact on Longevity of Genetic Cardiovascular Risk and Lifestyle including Red Meat Consumption

**DOI:** 10.1155/2020/1305413

**Published:** 2020-06-30

**Authors:** Alda Pereira da Silva, Maria do Céu Costa, Laura Aguiar, Andreia Matos, Ângela Gil, J. Gorjão-Clara, Jorge Polónia, Manuel Bicho

**Affiliations:** ^1^Ecogenetics and Human Health Unit, Genetics Laboratory, Environmental Health Institute-ISAMB, Faculty of Medicine, University of Lisbon, Av. Professor Egas Moniz, Piso 1C 1649-028 Lisboa, Portugal; ^2^Instituto de Investigação Bento da Rocha Cabral, Calçada Bento da Rocha Cabral 14, 1250-012 Lisboa, Portugal; ^3^CBIOS-Biosciences Research Center, School of Health Sciences and Technologies, Universidade Lusófona, Campo Grande 376, 1649-024 Lisboa, Portugal; ^4^IPLuso-ERISA/NICiTeS, Escola Superior de Saúde Ribeiro Sanches, Rua do Telhal aos Olivais, n8-8ª, 1900-693 Lisboa, Portugal; ^5^University Geriatric Unit, Environmental Health Institute-ISAMB, Faculty of Medicine, University of Lisbon, Portugal; ^6^Faculty of Medicine Oporto, Internal Medicine, Hypertension, Clinical Pharmacology, Alameda Prof. Hernâni Monteiro, 4200-319 Oporto, Portugal

## Abstract

**Background:**

Cardiovascular risk (CVR) underlies aging process and longevity. Previous work points to genetic and environmental factors associated with this risk.

**Objectives:**

The aim of this research is to look for any CVR gene-gene and gene-multifactorial/lifestyle interactions that may impact health and disease and underlie exceptional longevity.

**Methods:**

A case-control study involving 521 both gender individuals, 253 centenarians (100.26 ± 1.98 years), and 268 controls (67.51 ± 3.25 years), low (LCR, *n* = 107) and high (HCR, *n* = 161) CVR. Hypertension, diabetes, obesity (BMI, kg·m^−2^), and impaired kidney function were defined according to standard criteria. CVR was calculated using Q risk®. DNA was genotyping (*ACE*-rs4646994, *AGT*-rs4762, *AGR1*-rs5182, *GRK4*-rs2960306, *GRK4*-rs1024323, *NOS3-rs*1799983, and *SLC12A3*-rs13306673) through iPlex-MassARRAY®, read by MALDI-TOF mass spectrometry, and analyzed by EARTDECODE®.

**Results:**

Antilongevity factors consisted (OR 95% CI, *p* < 0.05) BMI 1.558 (1.445-1.680), hypertension 2.358 (1.565-3.553), smoking habits 4.528 (2.579-7.949), diabetes 5.553 (2.889-10.675), hypercholesterolemia 1.016 (1.010-1.022), and regular consumption of red meat 22.363 (13.987-35.755). Genetic aspects particularly for HCR individuals *ACE* II (OR: 3.96 (1.83-8.56), *p* < 0.0001) and *NOS3* TT (OR: 3.11 (1.70-5.70), *p* < 0.0001) genotypes were also risk associate. Obesity, smoking, hypercholesterolemia, and frequent consumption of red meat have an additive action to hypertension in the longevity process. There was a synergistic interaction between the endothelial *NOS3* genotypes and the severity of arterial hypertension. An epistatic interaction between functional genetic variants of *GRK4* and angiotensinogen was also observed.

**Conclusions:**

Cardiovascular risk-related genetic and multifactorial or predominantly lifestyle aspects and its interactions might influence the aging process and contribute to exceptional longevity in Portuguese centenarians. Besides lifestyle, the activity of nitrite oxide synthase may be one of the main physiologic regulators of cardiovascular protection in the path of longevity.

## 1. Introduction

The lifespan has been increasing in most countries, including Portugal. Particularly, the number of centenarians (CENT) in Portugal has almost tripled over the last 10 years from 589 in 2001 [1] to 1526 in 2011 [2], according to the last census. This value is estimated to be increasing in mainland Portugal, a number of individuals aged 100 years or over of 3342 in 2050 and 21177 individuals in 2080, mostly female, according to a central scenario [3].

Although in 2019 there were more CENT individuals and life expectancy has increased before COVID-19 pandemics, the estimated 65-year-old prospect of reaching 100 years old is still extremely low, i.e., over 17.6 years for men and 20.9 years for women, not reaching, in any case, the 100-year-old [4, 5].

Environmental factors, as well as genetic factors, all contribute to exceptional or extreme longevity that means over 99 years [6] or above 95 years, namely, in the case of male gender [7–9]. The contribution of genetic factors for longevity seems no higher than 35% [10]. This presupposes an epigenetic and lifestyle influence that has to be considered. There are many theories that try to explain the phenomenon of longevity and why an individual grows old. Nowadays, the combined theories have stood out and there is full awareness that biophysical, biochemical, and genetic factors may interact in the process [11, 12], in a highly complex matrix, regulated through systems and levels of organization of different complexities, which are intercommunicated through feedback systems [13]. Environmental and genetic agents, as well as their interaction, may lead to mechanisms related to oxidant stress and gene expression and influence the longevity process [14].

It is known that cardiovascular diseases have a great impact on achieving longevity since they constitute a risk for the shortening of life expectancy. The lifestyle associated with extreme longevity has been investigated; the hypothesis being raised of the influence of epigenetic factors on longevity and that genetic factors related to longevity may protect against the harmful effects of poor lifestyle choices [8]. Some genes linked to the aging process have been studied. Polymorphic genetic variants, related to the pathways involving aging and atherosclerosis [15], such as the Renin-Angiotensin-Aldosterone System (RAAS), message transduction, and oxidative stress, may affect the aging process, being susceptible to epistatic interactions, likewise with environmental factors. The insertion (I)/deletion (D) polymorphism of Angiotensin-Converting Enzyme (*ACE*) has been associated with longevity [16] and chronic diseases, such as CVDs and Alzheimer's dementia [15]. Other genes, such as angiotensinogen *(AGT*), specifically the *AGT* M235T polymorphism, may be associated with chronic age-associated CVDs [15]. As for *NOS3*, this is a determinant polymorphism for the production of nitric oxide, with consequent implications for the development of atherosclerosis, hypertension [16, 17], and Alzheimer's, with impact on cardiovascular risk and longevity [16, 18–20]. Variants of the AT1 receptor of angiotensin II (*AGTR1*) [21] and other genetic polymorphisms such as the G protein-coupled receptor kinase 4 (GRK4) [22, 23] and solute carrier family 12 member 3, SLC12A3 [24], have been related to arterial hypertension and atherosclerosis.

Cardiovascular risk (CVR) underlies aging process and longevity. Arterial hypertension (HT) and its severity are one of the main factors associated with cardiovascular risk. Previous work has shown that prevalence of HT in Portuguese Centenarians (64.4%) is lower than among Portuguese population aged ≥65 years (74.9%) (*p* < 0.001) [25] and that Portuguese centenarians, when hypertensive, have arterial pressure values from low to moderate, majority of grade 1 and only 4.7% grade 3 [16]. On the other hand, there were also found differences in frequency genotype distribution between centenarians and controls concerning *ACE*-I/D-rs4646994 (*p* = 0.001) and *NOS3*-GT-rs1799983 (*p* < 0.0001) polymorphisms [16]. The aim of the present study is the analysis of genetic variables associated with longevity and their interaction with cardiovascular risk variables and lifestyle, contributing to the understanding of the phenomenon of aging and its prevention, to delay its process.

## 2. Methods

Arterial hypertension and grade classification criteria were defined according to the European Society of Hypertension (ESH)/European Society of Cardiology (ESC) [26] and determined using a validated Colson MAM BP3AA1-2 device [27]. Pulse pressure abnormal was considered when above 62.5 mmHg [28]. Diabetes and obesity (body mass index, BMI, kg·m^−2^) were established in agreement with WHO criteria: diabetes for Hgb A1C ≥ 6.5%, or FPG < 126 mg/dL; obesity classifications: underweight < 18.5 kg·m^−2^, normal weight 18.5–24.9 kg·m^−2^, preobesity 25.0–29.9 kg·m^−2^, and obesity ≥ 30 kg·m^−2^ [29, 30]. Impaired kidney function was considered when blood urea nitrogen > 60 mg/dL and/or serum creatinine concentration ≥ 1.5 mg/dL [31].

The control group was subdivided into low (LCR) and high (HCR) cardiovascular risk, according to the results obtained, by inserting the personal data of each individual in a program, Q risk® 2-2016 [32], consisting in a prediction algorithm based on age, gender, ethnicity, body mass index (BMI), past and present smoking habits, hypertension criteria, systolic blood pressure, total cholesterol/HDL-c ratio, presence of diabetes, atrial fibrillation, rheumatoid arthritis, chronic kidney disease (stage 4 or 5), atrial fibrillation, and a family history of angina or heart attack in a first-degree relative aged <60 years [33, 34]. A high cardiovascular risk (HCR) score was considered if Q risk® ≥20% or in case of a history of acute myocardial infarction, stroke, peripheral arterial disease, congestive heart failure, or acute pulmonary edema. Q risk, predicts predicts a risk of developing cardiovascular disease in the next ten years as a stroke or heart attack.

The number of centenarians in the family until the 3rd generation was questioned.

Food consumption data were collected by applying a semiquantitative food frequency questionnaire, based on a validated FFQ for a Portuguese population [35]. Regular consumption of red meat was considered weekly consumption and up to three times a day.

DNA extractions were performed from the buccal epithelium sample using the commercial Citomed kit: “Citogene®Buccal Kit” Ref. Buc-100. The evaluation of DNA concentration and quality was done using the Multiskan Go Microplate spectrophotometer (Thermo Scientific®). Genotyping was performed using a MicroChip DNA on a high-throughput platform using iPlex MassARRAY® technology from Agena Bioscience. The genotypes obtained were read by MALDI-TOF mass spectrometry. The different mass values of each generated PCR product were converted into genotype information. Genotyping data were analyzed using the EARTDECODE® software system from HeartGenetics. The following genetic variants were studied: Angiotensin-Converting Enzyme (*ACE*) I/D, (-/(289pbALU) intr16; Cr 17), (rs4646994); angiotensinogen (*AGT*; Cr 1) (rs4762); angiotensin II receptor 1 (*AGR1*; Cr 3) (rs5182); endothelial nitric oxide synthase (*NOS3*; Cr 7) (rs1799983); G protein-coupled receptor kinase (*GRK4*; Cr 4) (rs2960306) and (rs1024323); member 3 solutes carrier of the family 12 (*SLC12A3*; Cr 16) (rs13306673).

### 2.1. Statistical Analysis

The Pearson *χ*^2^ test was used for comparisons between groups. The logistic regression method was conducted to model the probability of an existing event such as centenarians or controls, centenarian or low- or high-risk controls. In this logistic regression model, a binary dependent variable (categorical) was considered. The independent variables were binary with two classes, coded by an indicator or continuous variables. For the study of gene-gene or epistatic and gene-environment interactions, the multifactor dimensionality reduction (MDR) method was used through the MDR software package [36]. This methodology allows to perceive interactions between genes or between these and environmental factors, which interact for a given phenotype. The results are presented in the form of dendrogram and circle graphs, with visualization of additive and nonadditive connections or interactions in the phenotype. Positive entropy values indicate synergistic or nonadditive interaction, and negative values indicate redundancy. The red and orange connections indicate synergistic interactions, the green and brown connections indicate independence or additivity, and the blue ones indicate redundancy [37]. Data were analyzed throughout MDR 3.0.2 (available at http://www.multifactordimensionalityreduction.org/) and IBM SPSS statistics version 24.0 being statistical significance defined as a *p* value < 0.05.

## 3. Results

### 3.1. Sample

The sample consisted of 521 centenarians (CENT) and controls (CONT), from both genders. Centenarians from all the regions of Portugal were identified, enrolled, and evaluated at their usual place of residence, having their recruitment been previously described [38]. They consisted of 253 individuals with 100.26 ± 1.98 years old; from these, 197 were women (77.9%) mean age ± SD: 100.32 ± 1.95 years, and 56 were men (22.1%) mean age ± SD: 100.07 ± 2.12. The control group included patients recruited from the Heart and Vessels Department of Santa Maria Hospital, which is a reference hospital at the national level that receives patients from several regions of the country, and also from a Lisbon Health Care Center, in Portugal. The control group consisted of 268 individuals, with mean age 67.51 ± 3.25 years, being 165 women (61.6%) aged mean ± SD: 67.58 ± 3.19 years and 103 men (38.4%) with mean age ± SD: 67.41 ± 3.36. This sample was also the basis of other previous observational studies [16, 35, 38, 39]. This group was subdivided into low (LCR, *n* = 107) and high (HCR, *n* = 161) cardiovascular risk subgroups, based on Q risk®2-2016 score [33].

### 3.2. Cardiovascular and Lifestyle Risk Parameters

Regarding centenarian's sample individuals, 58.1% presented blood pressure levels within the normal range and the others, HT, being 27.3% grade 1, 12.6% grade 2, and 2% grade 3. From control sample individuals, 49.3% presented blood pressure levels within the normal range and the others, HT, being 31.7% grade 1, 13.4% grade 2, and 5.6% grade 3. There were no significant differences in the distribution of levels of blood pressure among genders in both subgroups (*p* = 0.336 and *p* = 0.720 CENT and CONT, respectively). It should be noted that these values refer to the observed blood pressure and include normotensive and hypertensive individuals, whether controlled or not.

Table 1 shows the results of sex-adjusted empirical analysis of covariates involved in cardiovascular risk and longevity, except for genetic variables. In this table, the OR and confidence intervals and their *p* value are compared, either taking as dependent variable CENT vs. LCR controls, either CENT vs. HCR controls, or CENT vs. CONT (control overall sample). The variables significantly (*p* < 0.001) most associated with the risk of not achieving exceptional longevity, considering CENT vs. CONT, expressed in OR (95% CI) were, in ascending order of risk, hypercholesterolemia 1.016 (1.010-1.022), LDL-c 1.017 (1.010-1.025), BMI 1.558 (1.445-1.680), hypertension 2.358 (1.565-3.553), smoking habits 4.528 (2.579-7.949), type 2 diabetes mellitus 5.553 (2.889-10.675), and frequent consumption of red meat 22.363 (13.987-35.755) (Table 1).

### 3.3. Genetic Parameters

#### 3.3.1. Centenarians in the Family

Considering the number of centenarian individuals in the family, it was verified that this prevalence is greater for the centenarians of the study sample, relative to the control group. Thus, while 34.6% of CENT have one or more centenarians in the family, the same occurs in only 19.9% of the controls (*p* = 0.015, *χ*^2^ = 10.450 gl = 3). Considering the linear-by-linear association, we find CENT vs. CONT (*p* = 0.002) with a centenarian relative: 27.9% vs. 17.3%, with 2 centenarians: 5.1% vs. 1.8%, and with 3 centenarian relatives: 1.5% vs. 0.9%.

#### 3.3.2. Genetic Polymorphisms and Cardiovascular Risk

The sex-adjusted empirical analysis of the genetic polymorphisms studied using different models (additive, recessive, and dominant) can be observed in Table 2. When considering the studied genotypes, significant differences were observed only in relation to *ACE* and *NOS3* genotypes, considering dependent variable centenarian's vs. controls (total, low risk, and high risk), being the referent and the covariates, the different genotypes depending on the model (Table 2). It was found that having the *ACE* II genotype increased 3.2 times the risk of not achieving longevity in low-risk controls (*p* = 0.004) and about 4 times in high-risk controls (*p* < 0.0001). Similarly, having the *NOS3* TT genotype increases 2.1 times the risk of not achieving longevity in low-risk controls (*p* = 0.021) and 3.1 times in high-risk controls (*p* < 0.0001) (Table 2).

### 3.4. Variable Interaction and Longevity

**Figure 1:**
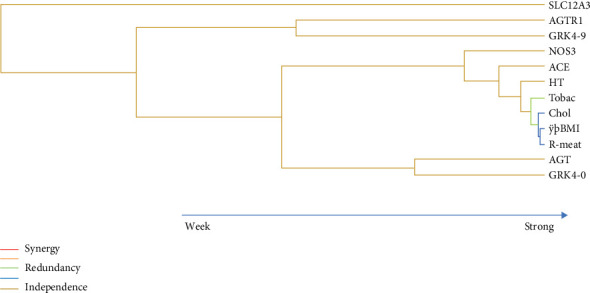
Dendrograms of interaction between the genetic variants studied and hypertension, smoking habits, hypercholesterolemia, BMI, and red meat intake, in the process of longevity (CENT and CONT). The shorter the line connecting two attributes, the stronger means the interaction.

Through the reduction of multifactorial dimensionality, it can be verified that there is a close and strong genotype interaction between *ACE* and *NOS3* that in turn relates to hypertension as well as to hypercholesterolemia, smoking, and red meat intake (Figure 1).

**Figure 2:**
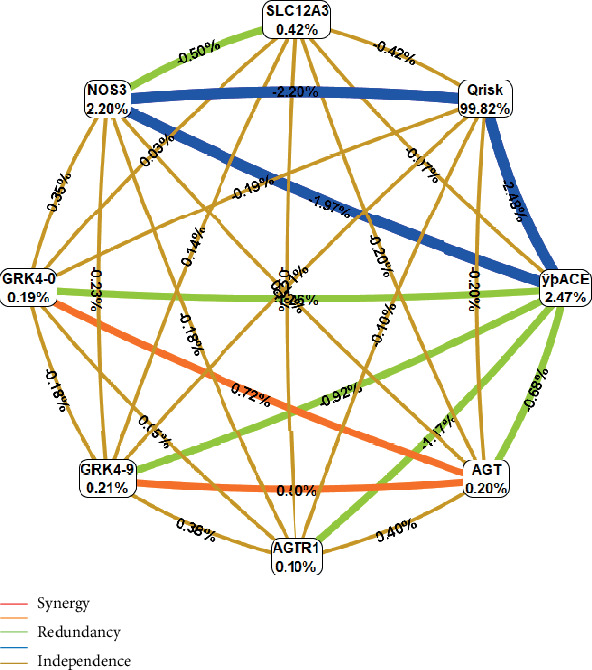
Circular graph of genotype interaction between cardiovascular risk genes and Q risk in the process of longevity. Q risk: calculated prediction algorithm based on age, gender, ethnicity, BMI, past and present smoking habits, systolic blood pressure level, total cholesterol/HDL-c ratio, presence of hypertension, diabetes, atrial fibrillation, rheumatoid arthritis, chronic kidney disease (stage 4 or 5), and a family history of angina or heart attack in a first-degree relative aged <60 years.

When considering the interactions of the genetic variants studied with multifactorial parameters related to cardiovascular risk isolated or determined as Q risk, there is a predominant interaction between this marker and genetic polymorphisms *NOS3* GT and *ACE* I/D related to exceptional longevity (Figure 2).

**Figure 3:**
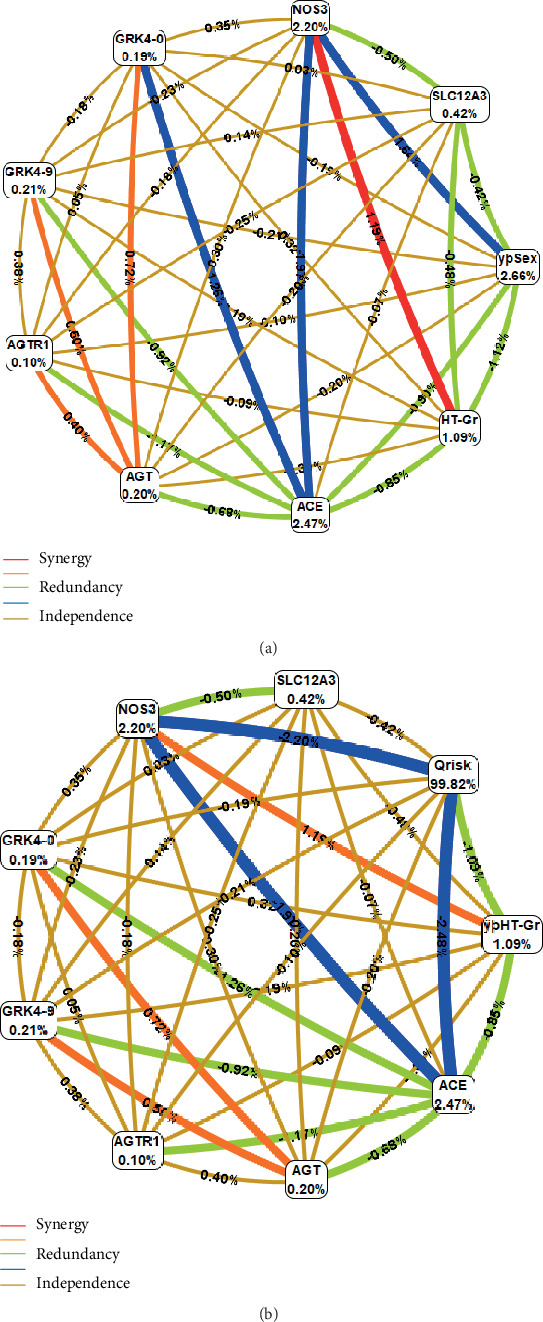
Circular graph of genotype interaction between cardiovascular risk genes with sex and hypertension grade (a) or with Q risk and hypertension grade (b), in the process of longevity.

Since the grade of hypertension is one of the most prominent cardiovascular risk factors, we looked to see if the genetic variants studied could interact with hypertension severity. Although there were no differences in *NOS3* genotype distribution according to the levels of blood pressure in both subgroups CENT *p* = 0.554 and CONT *p* = 0.069, a high degree of synergy was detected between hypertension grade and *NOS3* genotype and also, an epistatic interaction although less strong than the latter was observed between *ANG* and *GRK4* genotypes for longevity (Figure 3(a)). When including Q risk in the model, the same observations are verified (Figure 3(b)).

## 4. Discussion

The underpinnings underlying the natural aging process are complex, given their multifactorial nature. This work is aimed at contributing to understanding the relative importance of some CVR genetic as well as lifestyle aspects in the approach to exceptional longevity. It was not surprising that aspects such as diabetes, obesity, hypercholesterolemia, hypertension, and smoking contradict extreme longevity (Table 1). Moreover, it was noticed how these aspects interact with each other and with the genotypes. When analyzing the graphs, it is noticed that there is a redundant or additive interaction between variables such as red meat consumption, hypercholesterolemia, obesity, smoking, and high blood pressure, which adds to the genetic aspects along the path of longevity (Figure 1). Smoking has been associated with all major dead causes as cardiovascular and respiratory diseases and has been associated with more than 7 million deaths per year [40], a value far superior than diabetes, which has been related to 1.6 million deaths worldwide [41]. Our findings support this because the frequency of smoking habits in centenarians was low (8.7%) and diabetes (4.7%) was rare, which differed significantly from those in the control group (heavy smokers 20.3% and diabetics 22.0%). The majority of centenarians (91.3%) never smoked, and when they did, it was in the amount of less than one pack per year, which did not happen in the control group in which 20.3% of individuals reported smoking more than one pack per day.

Hypertension is the main risk factor for CV disease. It was therefore named “the number one killer” by the WHO [42]. However, there are hypertensive centenarians and HT per se does not seem to prevent reaching a well above average age [43, 44]. Thus, the interference of factors other than HT may contribute to longevity. The present study shows a frequency of HT in the Portuguese centenarians (64.4%), similar to that found among centenarians in other European countries such as Poland (65.0%) [44] and Spain (64.0%) [45]. It should be pointed out that the prevalence of hypertension in Portuguese centenarians was significantly lower (*p* < 0.0001) than in a large national study on hypertension, according to which 74.9% of individuals over 64 years are hypertensive [25]. In another study conducted in the Portuguese population over 55 years of age, the prevalence of HT was 67.6% according to the JNC VI criterion [46].

The probable justification for hypertensive individuals to reach the age of 100 years may be the fact that, although hypertensive, they have controlled arterial pressures. In fact, there were significant differences regarding the control of hypertension: while the frequency of controlled hypertensive individuals verified in the study concerning the Portuguese hypertensive population was 42.5% [25], in relation to the studied centenarians, the frequency of controlled hypertension was higher (58.9%) (*χ*^2^ = 16.03, *p* < 0.0001). In addition, centenarians, when hypertensive, have low to moderate blood pressure values and most have grade 1 hypertension [16]. Increased pulse pressure has been shown to be an independent cardiovascular risk factor, especially for very elderly individuals [47]. Our data show that pathological pulse pressure was prevalent in the CENT in relation to CONT (*p* < 0.030) although the mean PP found in individuals at HCR did not differ significantly from those found in the studied CENT. This means that HCR individuals approach centenarians in terms of cardiovascular aging (Table 1).

Individuals in the centenarian group had lower total cholesterol, LDL-c, non-HDL cholesterol, and total cholesterol/HDL-c ratio [35]. LDL-c and non-HDL are atherogenic factors. Non-HDL cholesterol includes triglyceride-rich lipoproteins, remaining TG-rich lipoproteins enriched with cholesteryl esters and also lipoprotein (a), and has a high CV risk predictive value [48]. In relation to the cholesterol/HDL-c ratio, one of the CVR parameters, the centenarians had a better profile in particular when compared with the subjects in the high-risk subgroup of the control group [35]. In addition, centenarian individuals showed a significantly lower LDL mean value relative to the control group [35]. These aspects may be associated with their longevity success, and eating habits underlie this profile. In fact, membrane-rich cholesterol domains (lipid rafts) in which the receptors aggregate play an important role in signaling mechanisms, and activation of NADPH oxidase leading to an increase of oxidant stress in the cell which may induce LDL-c modification [49]. In addition to the lowest LDL-c, the centenarians present the total cholesterol/HDL-c indicator (used to calculate Q risk), which is significantly lower in relation to the high-risk subgroup of the control group [35]. This aspect is relevant since HDL particles remove fat molecules from cells such as cholesterol (especially oxidized), phospholipids, and triglycerides protecting arteries' walls [50]. Moreover, HDL receptors, such as ABCA1 and SR-BI, may act as anti-inflammatory receptors [51]. The inflammatory process, by stimulating the production of the acute phase protein hepcidin, leads to the internalization of its receptor, the ferroportin channel, in macrophages, preventing iron exporting from these cells, with resultant accumulation of iron. This phenomenon will contribute to the production of reactive intracellular oxygen species, leading to lipid oxidation preventing their efflux from inside the cell, facilitating the process of lipoperoxidation and modification of LDL, contributing to atheroma formation in a vicious cycle. This inflammatory condition generates more radicals that perpetuate this vascular process generalizing atherosclerosis [52].

Not only diet [53] but obesity [54] may accelerate the aging process. Low red meat intake was, in the present work, also checked for longevity impact (Table 1). Our results agree with that observed by other authors suggesting that the highest CVR were associated with the highest frequencies of red meat consumptions [35, 55, 56]. Particularly, processed red meat is associated with a higher incidence of CV diseases such as coronary heart disease, heart failure, and stroke in addition to other pathologies [56]. Red meat is a source of heme-iron [57] which, besides carcinogenesis pathology [58, 59] in the presence of an inflammatory process, facilitates LDL lipoperoxidation and atherogenesis [52]. As the low consumption of red meat throughout life, the normal weight and the healthy level of visceral fat [38] were prevalent among Portuguese centenarians; the results of the present study confirm the evidence that the lifestyle certainly contributed to their low cardiovascular risk and longevity (Table 1).

Regarding the genetic aspects, the contribution of genetics for longevity is estimated as 5 to 35% [10], thus paving the way to a wide range of environmental factors [6]. Actually, considering the number of centenarian individuals in the family, it is verified that this prevalence is greater for the CENT of the study sample, relative to the CONT group. Thus, while 34.6% of CENT have one or more centenarians in the family, the same occurs in only 19.9% of the controls. The genetic influence, minimum before age 60, increases thereafter, becoming genetic factors increasingly important to achieve longevity [60]. Exceptional longevity may be influenced by polymorphisms in specific genes, coupled with superior genomic stability and homeostatic mechanisms [6]. Despite a genetic association to longevity, the environment can influence gene expression through possible epigenetic mechanisms [61], with lifestyle factors being a major contributor to longevity.

The fact that the number of centenarians increases, according to the Demographic Statistics Unit of the Portuguese National Statistics Institute [3], corroborates the environmental influence in contributing to longevity, which is in line with other authors [6, 55, 62].

Studies indicate the existence of genetic interactions between renin-angiotensin system loci [63, 64] and endothelial nitric oxide synthase polymorphisms [65]. In the present work, interactions of different types and intensity were verified, among *GRK4*, *ACE*, *NOS3*, *AGT*, and *AGR1* gene polymorphisms (Figures 1 and 2).

The *GRK4* gene polymorphism may, in synergy with that of the angiotensinogen gene, influence CVR (Figure 2). Polymorphic variants of angiotensinogen gene (M235T, rs699, and T174M, rs4762) could be associated with angiotensinogen levels, HT, left ventricular hypertrophy, and survival in heart failure [66]. Polymorphisms of this protein may be on the basis of susceptibility to hypertension, including during pregnancy [67]. It is reported genetic interaction of genes related to the RAAS, namely, angiotensinogen with environmental factors, affecting longevity by conditioning susceptibility to HT [68]. An angiotensinogen derivative, i.e., angiotensin II, in turn, throughout renin and ACE activities, acts through angiotensin II type 1 receptor that plays an important role in vasoconstriction and retention of salts and water, which action is modulated by the associated *AGR1* CT genotype [69].

The strong redundant interactions of red meat consumption, smoking, BMI, and hypercholesterolemia are additive to high blood pressure, *ACE*, and *NOS3* genotype polymorphisms, all being able to contribute to cardiovascular risk. The question that arises is to what extent can the genotypic variants of these enzymes condition the harmful effects of these variables obstacle to longevity?

It should be noted that ACE plays a critical role in sodium and erythrocyte homeostasis. This enzyme catalyzes the formation of angiotensin II from angiotensinogen and inactivation of bradykinin, and goralatide, an inhibitor of hematopoiesis N-acetyl-seryl-aspartyl-lysyl-proline, resulting in increased vasoconstriction and blood pressure and appropriate erythropoiesis [70]. The insertion/deletion polymorphism (ID) has been studied in hypertensive individuals; the D allele is associated with higher plasma levels of ACE. The DD genotype is significantly associated with higher systolic and diastolic blood pressure [71–73]. However, the DD genotype may be advantageous in this group of centenarians in the absence of CV environmental risk factors (Table 2), leading to a favorable gene-environment interaction which may have contributed to longevity.

It is understood that genetic variants of *GRK4* (rs2960306 and rs1024323) may, in synergistic interaction with the angiotensinogen gene, influence CVR (Figures 2 and 3). The polymorphisms in the *GRK4* gene are associated with the etiology of arterial hypertension by regulating phosphorylation and the function of dopamine receptors in proximal renal tubule cells which may lead to a decrease in sodium elimination and a consequent increase in blood pressure [74–76]. Studies indicate that *GRK4* gene polymorphisms are associated with susceptibility to hypertension in the European population [22] and Caucasians [77]. Both angiotensinogen and G protein-coupled receptor kinase are ultimately related to sodium retention, so their epistatic relationship makes sense.

The human endothelial nitric oxide synthase has a heme domain [78, 79]. It is constitutively expressed, being a mediator of age-related phenotypes and longevity [80].

Nitric oxide (NO) product of the oxidation of L-arginine by NOS3 is a gaseous free radical, which can function as a signaling molecule that, through a process of nitrosylation, regulates a series of physiological responses such as oxygen consumption, insulin secretion, apoptosis, neurotransmission, immunity, vasodilation, and angiogenesis. For its vasoprotective and antiatherosclerotic proprieties, it regulates smooth muscle relaxation and interacts with mitochondria, triggering mechanisms of cell survival or death [17, 80, 81].

The endothelial *NOS3* (SNP rs1799983) (Cr.7q36.1) have a GT polymorphism guanine to thymine change in position 894 of the *NOS3* gene leading to Glu298Asp [82]. This polymorphism allows the generation of two isoforms of endothelial *NOS3*: *NOS3* Glu298 (allele G) and *NOS3* Asp298 (allele T) [83]. It is a functional polymorphism, which influences the activity of the enzyme, NO production, blood pressure [17, 84], lipid levels [85], and angiogenesis [86] with people with the T allele being particularly homozygous, associated with less enzyme activity [84] and the highest cardiovascular risk [18, 19, 85], reduced NO bioavailability engaging in the initiation, progression, and complications of atherosclerosis [87] less likely to achieve longevity [16]. These mechanisms can explain what was observed in the present study of the synergistic interaction of *NOS3* genotypes with the degree of HT in longevity (Figures 2 and 3) as well as the potentially additive effect of smoking, hypercholesterolemia, obesity, and frequent consumption of red meat (Figure 1). The risk associated with this consumption (Table 1) has been neglected in longevity studies, but it is important that it be deepened because red meat increases heme-iron levels, inducing lipid peroxidation products, generating an inflammatory process and endogenous formation of abnormal NO radical derivatives as nitration compounds, endogenous nitrosamines, also induced by tobacco, responsible for the known harmful effects of NO [88, 89]. Several studies have demonstrated that nonhemodynamic factors such as chronic inflammation and oxidative stress may contribute to Early Vascular Aging (EVA).

On the other hand, caloric restriction may reverse this process and promote Healthy Vascular Aging (HVA) or even Super Normal Vascular Aging (SUPERNOVA) [90]. These data may lead to a reflection on the importance of eating habits such as caloric overload and in particular that associated with red meat ingestion in longevity [35].

## 5. Conclusions

This study realized and confirmed some of the cardiovascular risk factors, both genetic and related to lifestyle, and their interaction, in longevity. It concludes that obesity, hypercholesterolemia, and hypertension, according to their severity and smoking and consumption of red meat according to their frequency of consumption, are aspects to control when trying to follow the path of longevity. These factors interact with each other, in a redundant and additive way to genetic factors, creating the long-lived phenotype. Genetic polymorphisms associated with increased cardiovascular risk, such as *ACE* and *NOS3*, can modulate lifestyle aspects.

Being centenarian, it is a result of a set of life circumstances, environmental factors in their broadest concept associated with a genetic context that converged in the prevention of cardiovascular risk factors, translating into a phenotype of exceptional longevity. According to our results, being centenarian is strongly determined by environmental/lifestyle factors, specifically lack of smoking and nonregular red meat consumption habits and, to a lesser extent, eutrophic phenotype, low grade, and controlled hypertension when existent, adequate lipid profile. Thus, interactions between genetic variants of polymorphisms with hemodynamic regulation in particular of *ACE* and *NOS3* with environmental factors may contribute to exceptional longevity. Functional genetic variants of the GRK4 enzyme may, as evidenced, in synergy with those of the angiotensinogen gene, influence CVR and longevity. This synergism shows the relevance of the coordinated hepatorenal action in longevity. Also, the synergistic interaction of the NOS3 endothelial enzyme genotypes, conditioning the activity of this enzyme, with the degree of arterial hypertension, denotes to be one of the relevant mechanisms in the microcirculatory preservation in longevity. Future studies may see the extent to which individuals have protective factors, either genetic or lifestyle, that allow longevity and healthy aging.

## 6. Study Limitations

In this study, the group of centenary individuals is compared to a control group of elderly, but younger, in order to ensure control of risk factors, assuming that the probability of reaching 100 years is remote, according to the bank contemporary database of Portugal [4]. In addition, it is assumed that the cardiovascular risk of centenarians is small compared to the high-risk group in the control group. In fact, otherwise, they would not have reached such a high age.

## Figures and Tables

**Table 1:** Empirical analysis gender adjusted (univariate logistic regression) of covariates involved in cardiovascular risk and longevity.

Variables in empirical analysis	CENT vs. LCR		CENT vs. HCR		CENT vs. CONT	
	^¥^OR (CI 95)	*p* value	^¥^OR (IC95)	*p* value	^¥^OR (CI 95)	*p* value
BMI^∗^	1.517 (1.386-1.660)	**<0.0001**	1.581 (1.443-1.732)	**<0.0001**	1.558 (1.445-1.680)	**<0.001**
SBP^∗^	1.007 (0.996-1.018)	0.215	1.027 (1.016-1.038)	**<0.0001**	1.018 (1.010-1.027)	**<0.001**
DBP^∗^	1.076 (1.051-1.101)	**<0.0001**	1.084 (1.062-1.108)	**<0.0001**	1.084 (1.065-1.105)	**<0.001**
MBP^∗^	1.045 (1.025-1.065)	**<0.0001**	1.068 (1.048-1.088)	**<0.0001**	1.060 (1.043-1.076)	**<0.001**
HT	1.096 (0.677-1.772)	0.710	6.369 (3.272-12.396)	**<0.0001**	2.358 (1.565-3.553)	**<0.001**
PP	0.384 (0.238-0.619)	**<0.0001**	1.154 (0.750-1.775)	0.515	0.719 (0.506-1.021)	0.065
Diabetes	—	0.999	16.252 (7.969-33.141)	**<0.0001**	5.553 (2.889-10.675)	**<0.001**
Cholesterol T^∗^	1.018 (1.010-1.026)	**<0.0001**	1.012 (1.006-1.019)	**<0.0001**	1.016 (1.010-1.022)	**<0.001**
HDL-c^∗^	1.054 (1.028-1.081)	**<0.0001**	1.011 (0.988-1.033)	0.350	1.034 (1.014-1.055)	**0.001**
Triglycerides^∗^	0.993 (0.986-1.001)	0.076	1.008 (1.001-1.015)	**0.023**	1.001 (0.996-1.006)	0.680
LDL-c^∗^	1.019 (1.010-1.029)	**<0.0001**	1.014 (1.006-1.022)	**0.001**	1.017 (1.010-1.025)	**<0.001**
Cholesterol/ HDL-c^∗^	0.968 (0.735-1.274)	0.816	1.390 (1.047-1.843)	**0.023**	1.156 (0.915-1.460)	0.225
Tobacco	1.777 (0.750-4.210)	0.191	6.180 (3.415-11.185)	**<0.0001**	4.528 (2.579-7.949)	**<0.001**
Regular red meat consumption	14.874 (8.380-26.401)	**<0.001**	31.636 (17.247-58.028)	**<0.001**	22.363 (13.987-35.755)	**<0.001**

^∗^Analyzed as continuous variables; CENT: centenarians; LCR: low cardiovascular risk; HCR: high cardiovascular risk; CONT: controls (LCR plus HCR); BMI: body mass index; SBP: systolic blood pressure; DPP: diastolic blood pressure; MBP: mean blood pressure; PP: pulse pressure. All parameters adjusted to sex, taking as a reference to the female gender. ^¥^Considering the dependent variable to be CENT versus CONT of high (HCR) or low (LCR) cardiovascular risk, the Odds Ratio (OR) refers to the measure of association between the variables in empirical analysis and the risk of not being centenary.

**Table 2:** Gender-adjusted empirical analysis of genetic polymorphisms (covariates) potentially involved in longevity and associated with cardiovascular risk.

SNPs	CENT vs. LCR		CENT vs. HCR		CENT vs. CONT	
	OR (95 CI)	*p*	OR (95 CI)	*p*	OR (95 CI)	*p*
*ACE* (ID) rs4646994						
*Additive model*						
DD	Referent		Referent		Referent	
ID	0.96 (0.58-1.61)	0.884	2.72 (1.29-5.74)	**0.009**	1.23 (0.83-1.81)	0.302
II	3.16 (1.44-6.93)	**0.004**	3.96 (1.83-8.56)	**<0.0001**	3.43 (1.77-6.64)	**<0.0001**
*Recessive model*						
DD	Referent		Referent		Referent	
II/ID	1.22 (0.75-1.97)	0.420	1.74 (1.10-2.76)	**0.018**	1.49 (1.03-2.16)	**0.036**
*Dominant model*						
ID/DD	Referent		Referent		Referent	
II	3.22 (1.54-6.76)	**0.002**	3.20 (1.56-6.58)	**0.002**	3.07 (1.64-5.73)	**<0.0001**
*AGT* (CT), *CM920009* rs699						
*Additive model*						
CC	Referent		Referent		Referent	
CT	1.61 (0.78-3.46)	0.196	0.63 (0.29-1.39)	0.255	0.98 (0.55-1.76)	0.951
TT	—	0.999	3.55 (0.45-28.09)	0.229	1.99 (0.27-14.71)	0.499
*Recessive model*						
CC/CT	Referent		Referent		Referent	
TT	—	0.999	3.82 (0.49-29.97)	0.203	2.0 (0.27-14.71)	0.496
*Dominant model*						
CC	Referent		Referent		Referent	
CT/TT	1.55 (0.74-3.26)	0.248	0.75 (0.36-1.55)	0.433	1.03 (0.58-1.81)	0.927
*AGT* (TC), *CM920010* rs4762						
*Additive model*						
TT	Referent		Referent		Referent	
TC	1.53 (0.86-2.72)	0.149	0.74 (0.46-1.20)	0.218	0.98 (0.65-1.47)	0.915
CC	1.94 (0.94-3.99)	0.071	0.91 (0.49-1.71)	0.773	1.178 (0.69-2.00)	0.546
*Recessive model*						
TT/TC	Referent		Referent		Referent	
CC	1.46 (0.80-2.67)	0.216	1.08 (0.61-1.91)	0.785	1.19 (0.75-1.90)	0.460
*Dominant model*						
TT	Referent		Referent		Referent	
TC/CC	1.62 (0.94-2.82)	0.085	0.78 (0.50-1.23)	0.285	1.03 (0.70-1.51)	0.890
*AGR1* (CT) rs5182						
*Additive model*						
CC	Referent		Referent		Referent	
CT	1.13 (0.66-1.92)	0.655	0.98 (0.61-1.59)	0.945	1.06 (0.71-1.59)	0.763
TT	0.99 (0.51-1.93)	0.978	0.97 (0.52-1.79)	0.920	1.01 (0.61-1.67)	0.977
*Recessive model*						
CC/CT	Referent		Referent		Referent	
TT	0.92 (0.51-1.66)	0.789	0.98 (0.56-1.71)	0.937	0.97 (0.62-1.54)	0.908
*Dominant model*						
CC	Referent		Referent		Referent	
CT/TT	1.09 (0.66-1.79)	0.748	0.98 (0.63-1.53)	0.926	1.05 (0.72-1.52)	0.812
*NOS3* (GT) rs1799983						
*Additive model*						
GG	Referent		Referent		Referent	
GT	1.19 (0.70-2.04)	0.517	1.35 (0.81-2.24)	0.256	1.28 (0.85-1.94)	0.242
TT	2.14 (1.12-4.10)	**0.021**	3.11 (1.70-5.70)	**<0.0001**	2.78 (1.66-4.64)	**<0.0001**
*Recessive model*						
GG	Referent		Referent		Referent	
TT/GT	1.44 (0.88-2.36)	0.148	1.80 (1.13-2.87)	**0.013**	1.66 (1.14-2.43)	**0.009**
*Dominant model*						
GT/GG	Referent		Referent		Referent	
TT	1.95 (1.09-3.48)	**0.024**	2.66 (1.55-4.55)	**<0.0001**	2.43 (1.53-3.87)	**<0.0001**
*GRK4, CM025429* rs2960306						
*Additive model*						
GG	Referent		Referent		Referent	
GT	1.07 (0.64-1.80)	0.801	0.74 (0.46-1.19)	0.211	0.89 (0.60-1.32)	0.556
TT	1.31 (0.66-2.60)	0.446	0.69 (0.35-1.38)	0.295	0.97 (0.56-1.68)	0.967
*Recessive model*						
GG/GT	Referent		Referent		Referent	
TT	1.26 (0.68-2.34)	0.469	0.81 (0.43-1.54)	0.521	1.03 (0.62-1.72)	0.908
*Dominant model*						
GG	Referent		Referent		Referent	
GT/TT	0.89 (0.54-1.45)	0.632	0.73 (0.47-1.14)	0.162	0.91 (0.63-1.32)	0.607
*GRK4, CM025430 rs1024323*						
*Additive model*						
CC	Referent		Referent		Referent	
CT	1.24 (0.73-2.12)	0.427	0.73 (0.45-1.17)	0.190	0.92 (0.62-1.37)	0.694
TT	1.75 (0.87-3.49)	0.115	0.81 (0.40-1.61)	0.541	1.14 (0.65-1.99)	0.650
*Recessive model*						
CC/CT	Referent		Referent		Referent	
TT	1.54 (0.83-2.83)	0.171	0.95 (0.50-1.82)	0.880	1.19 (0.71-1.98)	0.507
*Dominant model*						
CC	Referent		Referent		Referent	
CT/TT	1.36 (0.82-2.24)	0.237	0.75 (0.48-1.17)	0.197	0.97 (0.67-1.41)	0.877
*SLC12A3* rs13306673						
*Additive model*						
CC	Referent		Referent		Referent	
CT	0.80 (0.39-1.63)	0.534	1.40 (0.75-2.58)	0.288	1.09 (0.65-1.83)	0.746
TT	—		—	0.999	—	0.999
*Recessive model*						
CC/CT	Referent		Referent		Referent	
TT	—		—	0.999	—	0.999
*Dominant model*						
CC	Referent		Referent		Referent	
CT/TT	0.80 (0.39-1.63)	0.534	1.47 (0.80-2.69)	0.210	1.14 (0.68-1.90)	0.629

LCR: low cardiovascular risk; HCR: high cardiovascular risk; CENT: centenarians; CONT: controls.

## Data Availability

The data that support the findings of this study are available from the corresponding author upon request.

## Abbreviations

*ACE*: Angiotensin-Converting Enzyme
*AGT*: Angiotensinogen
*AGR1*: *A*ngiotensin II receptor 1
BMI: Body mass index
CENT: Centenarians
CONT: Controls
CVR: Cardiovascular risk
DBP: Systolic blood pressure
ESC: European Society of Cardiology
ESH: European Society of Hypertension
*GRK4*: G protein-coupled receptor kinase
HCR: High cardiovascular risk
HT: Arterial hypertension
LCR: Low cardiovascular risk
MBP: Mean blood pressure
MDR: Multifactor dimensionality reduction
*NOS3*: Endothelial nitric oxide synthase
RAAS: Renin-Angiotensin-Aldosterone System
SBP: Systolic blood pressure
*SLC12A3*: Member 3 solutes carrier of the family 12.
